# Hemodiafiltration combined with polymyxin B-immobilized fiber column direct hemoperfusion is effective for acute postoperative exacerbation of interstitial pneumonia: a case report

**DOI:** 10.1186/s40981-022-00589-2

**Published:** 2022-12-28

**Authors:** Tatsuro Yokoyama, Takahiro Tamura, Harunori Nakashima, Morihide Ando, Koshiro Kikkawa, Ryohei Ito

**Affiliations:** 1 Department of Anesthesiology, Ogaki Municipal Hospital, Ogaki, Japan; 2 Department of Anesthesiology, Nagoya University Graduate School of Medicine, Nagoya, Japan; 3 Department of Respiratory Medicine, Ogaki Municipal Hospital, Ogaki, Japan

**Keywords:** Blood purification therapy, Interstitial pneumonia, Acute exacerbation, Extracorporeal membrane oxygenation, ECMO, Respiratory failure, Rheumatoid arthritis

## Abstract

**Background:**

Postoperative acute exacerbation of interstitial pneumonia has a high mortality rate; however, its treatment methods have not been standardized.

**Case presentation:**

A 72-year-old man with rheumatoid arthritis developed acute respiratory failure about 3 weeks after lung cancer surgery. There were increased diffuse frosted shadows in both lung fields. His condition was diagnosed as an acute exacerbation of interstitial pneumonia associated with rheumatoid arthritis, and he was started on steroid pulse therapy; however, his respiratory condition deteriorated. He was urgently intubated and started on veno-venous extracorporeal membrane oxygenation. Further, intensive care, including blood purification therapy, was initiated. The blood purification therapy comprised a combination of hemodiafiltration and 6-h polymyxin B-immobilized fiber column direct hemoperfusion. The patient was weaned off veno-venous extracorporeal membrane oxygenation, extubated, and discharged from the intensive care unit on the ninth day.

**Conclusions:**

Blood purification therapy was effective for acute exacerbation of interstitial pneumonia.

## Background

Postoperative acute exacerbation (AE) of interstitial pneumonia (IP) has high mortality rates [1]. A study showed that 83.3% of perioperative deaths resulting from pneumonia or acute respiratory distress syndrome among patients undergoing lung cancer resection are due to IP-AE [2]. However, there is no standard treatment for IP-AE [3]. The efficacy of polymyxin B-immobilized fiber column direct hemoperfusion (PMX-DHP) has been demonstrated in several patients with IP-AE refractory to steroid pulse therapy [4–6]. Furthermore, among patients with IPs, there have been reports of cases of IP-AE related to rheumatoid arthritis and AE of idiopathic pulmonary fibrosis [7]. This case report describes the management of postoperative IP-AE associated with rheumatoid arthritis, which could not be preoperatively diagnosed.

## Case presentation

A 72-year-old man (167 cm, 75 kg) had a history of rheumatoid arthritis, diabetes mellitus, and hypertension and a 51-year history of smoking 25 cigarettes/day. At the time of admission, the patient was taking baricitinib 4 mg and prednisolone 2 mg. He had a history of gastric ulcers and mild chronic anemia was noted. Thoracoscopic right upper lobectomy was planned because he was diagnosed as having right upper lobe lung cancer. Preoperative computed tomography (CT) findings were unremarkable; furthermore, respiratory function test results (%vital capacity, 118%; forced expiratory volume [FEV]1.0%, 72%; FEV1.0, 2.7 L; diffusing capacity for carbon monoxide [DLCO], 13.18 mL/min/mmHg; %DLCO, 72.5%) were within reference values. Moreover, the KL-6 value was 284 U/mL. Echocardiography revealed mild aortic regurgitation, moderate aortic stenosis, and mild mitral regurgitation; however, the patient showed preserved cardiac function with an ejection fraction of 65% and no history of heart failure. The patient underwent an unremarkable surgery, with a duration of surgery of 180 min, blood loss of 5 mL, urine volume of 240 mL, and transfusion volume of 1450 mL. Total intravenous anesthesia was administered. The duration of anesthesia was 245 min, and the duration of isolated lung ventilation was 188 min. During isolated lung ventilation, highly concentrated oxygen with an FiO_2_ of 60% to 100% and during both lung ventilation, an FiO_2_ of 40% were provided. The patient showed a good postoperative course and was discharged on the ninth postoperative day.

**Fig. 1:**
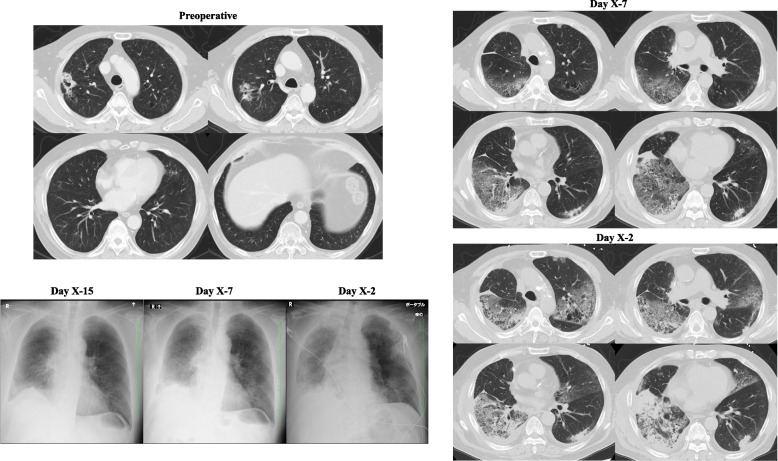
X-ray and computed tomography images of the patient’s chest before and after lung cancer resection. Preoperative examination revealed a lung cancer lesion in the right upper lobe. Examination upon postoperative readmission showed diffuse frosted shadows predominantly in the right lung (day X-7). However, upon intensive care unit (ICU) admission (day X-2), there were enlarged diffuse frosted shadows in both lung fields

On X-7, the 20th postoperative day, he returned to the outpatient department with a chief complaint of dyspnea, followed by hospitalization with a diagnosis of postoperative pneumonia. He was started on antibiotic therapy with piperacillin and tazobactam; however, there was no improvement and respiratory failure progressed. Radiography and CT revealed increased diffuse frosted shadows in both lungs (Fig. 1). These findings were indicative of IP-AE, which could not be preoperatively diagnosed.

Accordingly, steroid pulse therapy (methylprednisolone 1000 mg/day) was started on X-2. On day X, his respiratory condition further deteriorated; additionally, he was admitted to the intensive care unit (ICU) with clear consciousness; temperature, 37.5 °C; blood pressure, 121/60 mmHg; SpO_2_, 90% (FiO_2_, 80%); respiratory frequency, 36 times/min; and breathing with effort. Further, torsional hair sounds could be heard on the dorsal side without edema in the legs. The blood test results were as follows: aspartate, 85 U/L; alanine aminotransferase, 97 U/L; total bilirubin, 0.6 mg/dL; lactate dehydrogenase, 316 U/L; creatinine, 0.82 mg/dL; total protein, 6.1 g/dL; albumin, 1.9 g/dL; estimated glomerular filtration rate, 70.92 mL/min/1.73 m^2^; C-reactive protein, 9.21 mg/dL; white blood cell count, 32,710/μL; hemoglobin, 9.8 g/dL; hematocrit, 32.1%; platelet, 47.8 × 10^4^/μL; prothrombin activity, 35%; activated partial thromboplastin time, 46.2 s; fibrin degradation product, 9.1 μg/mL; antithrombin III, 81%; and KL-6, 245 U/mL.

The patient’s hemoglobin level was similar to that measured prior to the lung cancer resection and a mild coagulopathy of unknown cause was noted. He was urgently intubated due to respiratory distress; moreover, the findings of arterial blood gas analysis with FiO_2_ of 100% were as follows: pH, 7.348; pCO_2_, 41.9 mmHg; pO_2_, 47.6 mmHg; HCO_3_^−^, 22.5 mmol/L; sO_2_, 78%; and Lac, 24 mg/dL. Since the PaO_2_/FiO_2_ ratio was < 50, we started veno-venous extracorporeal membrane oxygenation (VV ECMO). Camostat mesylate was used for anticoagulation therapy with a target-activated clotting time of 160–180 s, without using heparin. Blood transfusion therapy was performed to maintain hemoglobin and fibrinogen levels at 12 g/dL and 200 mg/dL, respectively. The rate of morphine administration was adjusted to achieve a respiratory rate (RR) of 10–20 breaths/min. The ventilator settings were adjusted to match spontaneous breathing, with FiO_2_, 21%; PIP, 22 cmH_2_O; PEEP, 15 cmH_2_O; and ventilation volume ≤ 200 mL per cycle. Cyberestat sodium hydrate (300 mg/day) was started, followed by a combination of hemodiafiltration (HDF) and PMX-DHP. HDF was continuously performed using a polymethylmethacrylate hemofilter with a membrane area of 2.1 m^2^, dialysate flow rate (Qd) of 300 mL/min, filtration flow rate (Qf) of 1000 mL/h (post-dilution), and blood flow rate (Qb) of 150 mL/min. PMX-DHP was performed once for 6 h. Cyclophosphamide pulse therapy (900 mg/day) was administered on X + 1; additionally, post-treatment steroid pulse therapy (methylprednisolone 80 mg/day) was started. The patient was managed with dexmedetomidine, with a target score of 0 on the Richmond Agitation Sedation Scale. Furthermore, enteral feeding was started, and a physical therapist provided active physical therapy.

**Fig. 2:**
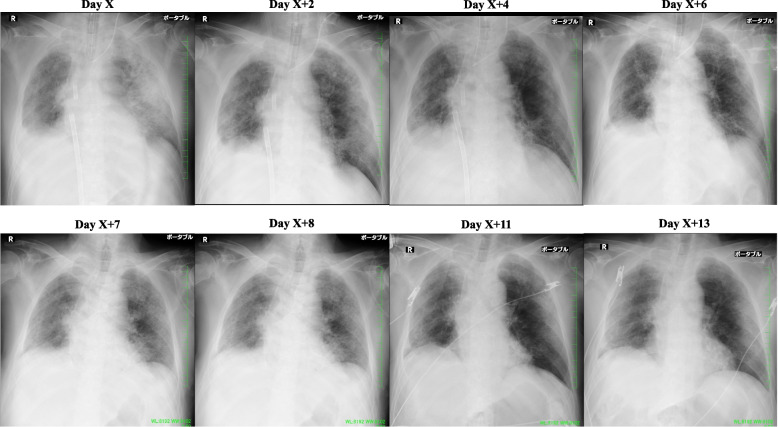
X-ray images showing the disease course of interstitial pneumonia in the patient after postoperative readmission to hospital. At the time of introduction of ECMO, bilateral frosted shadows and mild congestive images were detected, partly due to the effect of infusion. With the course of treatment, the frosted shadows tended to disappear and the congestive images improved. After extubation, a slight deterioration was observed, but the patient showed a tendency toward improvement (day X + 7)

The patient could communicate in writing and perform mobilization exercises except for the right hip joint and knee joint, where a devascularizing vessel was inserted. The scores of manual muscle strength tests for both upper limbs and the left lower limb were all 4 per 5. On X + 3, HDF was changed to continuous hemodiafiltration (CHDF; Qd, 300 mL/min; Qf, 300 mL/h; Qb, 150 mL/min) using a cytokine-adsorbing hemofilter. His body weight was reduced to ≈ 69.2 kg; additionally, he was maintained at a dry weight of 3 kg lower than the preoperative weight, with strict control of water intake and output. He showed mild and no edema of the dorsal hand surfaces and body trunk, respectively. Given the improved chest radiographs and increased ventilation volume without changes in the ventilator settings, we considered weaning VV ECMO (Fig. 2). The respiratory settings were PIP, 21 cmH_2_O; PEEP, 12 cmH_2_O; FiO_2_, 40%; and RR, 14 assisted breaths/min. In addition, the findings of the arterial blood gas test were as follows: pH, 7.442; pCO_2_, 44.5 mmHg; pO_2_, 104 mmHg; sO_2_, 97.8%; and HCO_3_^−^, 29.9 mmol/L.

After confirming that there were no changes in the respiratory status, including tachypnea or labored breathing, the patient was weaned from VV ECMO with simultaneous termination of CHDF. On X + 5, cyberestat sodium hydrate was discontinued and he could disengage and sit on the edge of the bed. Although he could not stably hold the end-sitting position independently, he could achieve it with light assistance. Steroid pulse therapy was readministered on X + 6. The ventilator settings were gradually reduced to PIP, 11 cmH_2_O; PEEP, 4 cmH_2_O; FiO_2_, 35%; SpO_2_, 96%; and RR, 16 times/min, with the patient being extubated at noon on X + 7. Two hours after extubation, he underwent a swallowing evaluation by a speech pathologist and started oral intake in the evening. SpO_2_ was maintained above 94% with nasal oxygen at rest at 3 L/min, with the patient being discharged from the ICU on X + 8. During the treatment course in the ICU, there were no significant findings in the blood, sputum, and urine culture tests as well as in the procalcitonin tests. The patient continued rehabilitation in the ward and was discharged on X + 23. At the 6-month follow-up, he lacked anxiety or depressive symptoms and had returned to work and driving, which indicated he did not have post-intensive care syndrome.

## Discussion

IP-AE is a highly fatal postoperative complication without an established treatment strategy [2, 3]. We successfully treated a case of IP-AE after lung cancer surgery through blood purification therapy.

We administered blood purification therapy involving a combination of HDF and PMX-DHP. The HDF was based on sustained high-efficiency daily diafiltration using a mediator-adsorbing membrane (SHEDD-fA) proposed by Nishida et al. [8]. SHEDD-fA may contribute to improved oxygenation during respiratory failure from sepsis; however, since this patient was on VV ECMO assistance, we could not assess whether autologous lung oxygenation was restored. Combined treatment with SHEDD-fA and PMX-DHP is effective for IP-AE [9], which is consistent with our findings. The patient did not respond to steroid therapy, his respiratory failure worsened, and he recovered after blood purification. Since his various culture test results were negative, it is unlikely that the antibiotics were effective, and it is also unlikely that the cyclophosphamide pulse therapy administered during the highly efficient hemodiafiltration procedure was sufficiently effective. However, based on the clinical course, we believe that the combination of SHEDD-fA and PMX-DHP was effective. Although we prioritized continuing HDF given its potential efficacy, early switching to CHDF should be considered in case the efficacy of antibiotic therapy and cyclophosphamide pulse is more important. We performed PMX-DHP for 6 h; however, some reports indicated superior outcomes when performed for ≥ 12 h [10]. We considered performing PMX-DHP twice [6]; however, the procedure was only performed once since the patient showed good progression without subsequent deterioration.

A limitation of our approach to this case is that the IP associated with rheumatoid arthritis was not preoperatively diagnosed. Instead, the diagnosis of IP-AE related to rheumatoid arthritis was made based on imaging findings and clinical course. Since IP was not preoperatively diagnosed, no special considerations were made in the anesthetic protocol for thoracoscopic right upper lobectomy. It is important to consider IP in cases involving an underlying collagen disease, including rheumatoid arthritis.

In conclusion, blood purification therapy was effective for IP-AE.

## Data Availability

Not applicable.

## Abbreviations

AE: Acute exacerbation
CT: Computed tomography
DLCO: Diffusing capacity for carbon monoxide
FEV: Forced expiratory volume
IP: Interstitial pneumonia
PMX-DHP: Polymyxin B-immobilized fiber column direct hemoperfusion
ICU: Intensive care unit
VV ECMO: Veno-venous extracorporeal membrane oxygenation
HDF: Hemodiafiltration
CHDF: Continuous hemodiafiltration
SHEDD-fA: Sustained high-efficiency daily diafiltration using a mediator-adsorbing membrane
RR: Respiratory rate
